# Supplementary material for: Molecular Phylogenetics and the Evolution of Morphological Complexity in Aytoniaceae (Marchantiophyta)
Source: Plants (Basel). 2024 Apr 9;13(8):1053. doi: 10.3390/plants13081053 (PMC11054525; doi:10.3390/plants13081053)
Supplement: Supplementary file 1 [file plants-13-01053-s001.zip › plants-2948960-supplementary.pdf]

Table S1 Sequences used in Aytoniaceae phylogenetic tree, including taxa, locations, vouchers, and GenBank accession numbers. Newly generated sequences in bold face. "---" missing data.

**Taxon**	**Voucher information**	**Localities**	**GenBank Accession**		
			***rbc*L**	***trn*L-F**	**nr 26S**
Asterella africana	*Schill & Lobos 29* (E)	Portugal	---	GQ910666	GQ910705
A. australis	*Glenny 6636* (?)	New Zealand	---	AF264660	---
A. bachmannii	*Duckett s.n.* (?)	Lesotho	---	AF264658	---
A. bolanderi	*Long 27554* (E)	USA	---	AF264657	---
***A. cruciata* I**	*Wei et al. 20141204-42* (HSNU)	China, Guangxi	**PP566135**	**PP566159**	**PP573773**
***A. cruciata* II**	*Peng et al. 20150130-2* (HSNU)	China, Yunnan	**PP566136**	**PP566160**	**PP573774**
***A. cruciata* III**	*Shu & Wei 20151012-7* (HSNU)	China, Sichuan	**PP566137**	**PP566161**	**PP573775**
***A. cruciata* IV**	*Bardunov s.n.* (KPABG)	Russia	---	KR024222	KR024186
***A. cruciata* V**	*Cao & Xiong 20141205-126* (HSNU)	China, Guizhou	**PP566138**	**PP566162**	**PP573776**
A. innovans	*Furuki 14274* (?)	USA, Hawaii	---	AF264656	---
***A. khasyana* I**	*Long 22420* (E)	India	---	AF264651	---
***A. khasyana* II**	*Xiang et al. 20161109-43* (HSNU)	China, Yunnan	**PP566134**	**PP566158**	**PP573772**
***A. khasyana* III**	*Zhu et al. 20150824-18* (HSNU)	China, Yunnan	**PP566141**	**PP566165**	**PP573779**
***A. khasyana* IV**	*Wang 20160813-1* (HSNU)	China, Yunnan	**PP566143**	**PP566167**	**PP573781**
A. lateralis	*Long 29598* (E)	Mexico	---	GQ910671	GQ910710
***A. leptophylla* I**	*Bakalin, P-40-24-12* (VBGI, KPABG)	Russia, Primorsky Territory	---	KR024221	KR024185
***A. leptophylla* II**	*Zhu & Promma 20160728-44* (HSNU)	China, Liaoning	**PP566142**	**PP566166**	**PP573780**
***A. leptophylla* III**	*Long & Furuki 24769* (?)	Japan	---	AF264652	---

***A. lindenbergiana* I**	*Long 19989* (E)	Norway	---	AF264653	---
***A. lindenbergiana* II**	*Konstantinova, K409-3-12* (KPABG)	Russia: Caucasus	---	KR024216	KR024180
***A. lindenbergiana* III**	*Konstantinova, K409-3-12* (KPABG)	Russia: Adygei	---	KR024219	KR024183
***A. lindenbergiana* IV**	*Konstantinova, K437-1-07* (KPABG)	Russia: Krasnodar	---	KR024218	KR024182
A. macropoda	*Gradstein 9728* (?)	Costa Rica	---	AF264655	---
***A. multiflora* I**	*Zhu et al. 20150824-44* (HSNU)	China, Yunnan	**PP566133**	**PP566157**	**PP573771**
***A. multiflora* II**	*Peng et al. 20150201-7* (HSNU)	China, Yunnan	**PP566144**	**PP566168**	**PP573782**
***A. multiflora* III**	*Zhu et al. 20150824-19* (HSNU)	China, Yunnan	**PP566145**	**PP566169**	**PP573783**
***A. multiflora* IV**	*Long 21637* (E)	Nepal	---	AF264654	---
***A. mussuriensis* subsp. *mussuriensis* I**	*Yin et al. 20160707-24* (HSNU)	China, Sichuan	**PP566146**	**PP566170**	**PP573784**
***A. mussuriensis* subsp. *mussuriensis* II**	*Xiang 20160916-9* (HSNU)	China, Yunnan	**PP566147**	**PP566171**	**PP573785**
***A. palmeri* I**	Doyle 11446 (E)	USA, California	KT793554	---	KT793342
***A. palmeri* II**	Long 27557 (E)	USA, California	---	AF264640	---
***A. palmeri* III**	Ma 17-9030 (KUN)	USA, California	**PP566125**	**PP566152**	**PP573767**
***A. saccata* I**	*Schill et al. 58* (E)	Switzerland, Valais	---	GQ910672	GQ910711
***A. saccata* II**	*Afonina s.n.* (LE, KPABG)	Russia, Chukotka	---	KR024220	KR024184
A. syngenesica	*Pócs 9415a* (?)	Réunion	---	AF264659	---
***A. tenella* I**	*Marsh 8995.3* (?)	USA., Arkansas	---	AF264650	---
***A. tenella* II**	*Goffinet 8750* (E)	USA, Connecticut	---	GQ910673	GQ910712
***A. wallichiana* I**	*Yamaguchi 11384* (?)	Japan, Iriomote	---	AF264648	---
***A. wallichiana* II**	*Yamaguchi 11280* (?)	Japan, Okinawa	---	AF264649	---
***A. wallichiana* III**	*Zanten 93.11.2334b* (?)	Philippines	---	AF264647	---

***A. wallichiana* IV**	*Wei & Shu 20151012-41* (HSNU)	China, Sichuan	**PP566148**	**PP566172**	**PP573786**
***A. wallichiana* V**	*Wei & Peng 20100913-112* (HSNU)	China, Guangxi	**PP566150**	**PP566174**	**PP573787**
***A. wallichiana* VI**	*Long 30251* (E)	Nepal, Lalitpur	---	GQ910674	GQ910713
***Asterellopsis grollei* I**	*Long 26964* (E)	China	---	AF264641	---
***Asterellopsis grollei* II**	*Long 27203* (E)	China, Qinghai	DQ286000	GQ910670	DQ265771
***Asterellopsis grollei* III**	*Xiang 20160925-10* (HSNU)	China, Yunnan	**PP566140**	**PP566164**	**PP573778**
***Asterellopsis grollei* IV**	*Zhu 20160815-21B* (HSNU)	China, Sichuan	**PP566139**	**PP566163**	**PP573777**
***Calasterella californica* I**	*Long 27651* (E)	USA	---	AF264639	---
***C. californica* II**	*Schill & Clarke 104* (E)	USA	---	GQ910667	GQ910720
***C. californica* III**	*Norris 80914* (?)	USA	---	AF228785	---
***Cryptomitrium himalayense* I**	*Xiang & Shen 20190908-25* (HSNU)	China, Sichuan	**PP566151**	**PP566175**	**PP573788**
***C. himalayense* II**	*Long 30559* (E)	Nepal, Rasuwa	---	GQ910678	GQ910717
C. tenerum	*Long 29748* (E)	Mexico, Querétaro	---	GQ910677	GQ910716
***Dumortiera hirsuta* I**	*Wang et al. 20140722-9* (HSNU)	China, Sichuan	KX792412	KX792398	---
***Dumortiera hirsuta* II**	*Cheng et al. 20150421-43* (HSNU)	China, Zhejiang	KX792414	KX792400	---
***Dumortiera hirsuta* III**	Wei et al. 20141204-44 (HSNU)	China, Guangxi	KX792413	KX792399	---
***Mannia androgyna* I**	*Schill & Lobos 32-2* (E)	Portugal, Madeira	---	GQ910679	GQ910718
***M. androgyna* II**	*Schill & Clarke 124* (E)	Namibia, Otjozondjupa	---	GQ910680	GQ910719
***M. californica* I**	*Schill & Clarke 164* (E)	India, Uttaranchal	---	GQ910681	GQ910720
***M. californica* II**	*Hugonnot s.n.* (E)	France, Ardèche	---	GQ910688	GQ910727
***M. californica* III**	*Xiang et al. 20160418-21* (HSNU)	China, Yunnan	**PP566126**	OR689868	OR700027
***M. controversa* subsp. *controversa* I**	*Köckinger s.n.* (E)	Austria, Carinthia	---	GQ910689	GQ910728

***M. controversa* subsp. *asiatica* II**	*Schill & Clarke 163* (E)	India, Uttaranchal	---	GQ910696	GQ910735
***M. fragrans* I**	*Schill & Clarke 162* (E)	India, Uttaranchal	---	GQ910690	GQ910729
***M. fragrans* II**	*Schill et al. 51* (E)	Switzerland, Valais	---	GQ910692	GQ910731
***M. fragrans* III**	*Itouga s.n.* (E)	Japan, Honshu	---	GQ910691	GQ910730
***M. fragrans* IV**	*Xiang 20160925-8* (HSNU)	China, Sichuan	**PP566127**	OR689869	OR700028
***M. gracilis* I**	*Hugonnot s.n.* (E)	France, Haute-Loire	---	GQ910669	GQ910708
***M. gracilis* II**	*Dulin MVD-1113* (KPABG)	Russia: Komi	---	KR024198	KR024172
***M. gracilis* III**	*Stefanut B3497* (E)	Romania, Bucegi	---	GQ910668	GQ910707
***M. pilosa* I**	*Schill et al. 84* (E)	Austria, Carinthia	---	GQ910693	GQ910732
***M. pilosa* II**	*Schill et al. 85* (E)	Austria, Carinthia	---	GQ910694	GQ910733
***M. pilosa* III**	*Dulin, s.n.* (KPABG)	Russia: Komi	---	KR024205	KR024175
M. sibirica	*Long & Schill 35669* (E)	USA, Minnesota	---	GQ910695	GQ910734
***M. triandra* I**	*Long & Schill 35668* (E)	USA, Minnesota	---	GQ910697	GQ910737
***M. triandra* II**	*Schill et al. 87* (E)	Austria, Carinthia	---	GQ910698	GQ910736
Plagiochasma appendiculatum	*Wei & Shu 20151012-53* (HSNU)	China, Sichuan	**PP566132**	**PP566156**	---
P. rupestre	*Schill 5* (E)	Portugal, Madeira	---	GQ910700	GQ910739
P. wrightii	*Long 29636* (E)	Mexico, Veracruz	---	GQ910701	GQ910740
***Reboulia hemisphaerica* I**	*Zhang et al. 20150830-182* (HSNU)	China, Fujian	**PP566128**	OR689873	OR700031
***R. hemisphaerica* II**	*Zhang et al. 20150830-181* (HSNU)	China, Fujian	**PP566149**	**PP566173**	---
***R. hemisphaerica* III**	*Shi 20150920-37* (HSNU)	China, Anhui	---	**PP566153**	**PP573768**
***R. hemisphaerica* IV**	*Borovichev BE13-6-07* (KPABG)	Russia, Murmansk	---	KR024212	KR024178
***R. hemisphaerica* V**	*Bakalin Kh-72-22-09* (VBGI, KPABG)	Russia, Perm Territory	---	KR024213	KR024179

***R. hemisphaerica* VI**	*Xiang 20160218-1* (HSNU)	China, Hunan	**PP566129**	OR689874	OR700032
***R. hemisphaerica* VII**	*Schill et al. 46* (E)	Switzerland, Valais	**---**	GQ910702	GQ910741
Targionia hypophylla	*Zhu et al. 20150824-32* (HSNU)	China, Yunnan	**PP566130**	**PP566154**	**PP573769**
Wiesnerella denudata	*Xiang et al. 20160418-6* (HSNU)	China, Yunnan	**PP566131**	**PP566155**	**PP573770**
